# Assessment of the dynamics of sensory perception of Wagyu beef strip loin prepared with different cooking methods and fattening periods using the temporal dominance of sensations

**DOI:** 10.1002/fsn3.1205

**Published:** 2019-09-26

**Authors:** Genya Watanabe, Michiyo Motoyama, Kazue Orita, Keigo Takita, Tatsuya Aonuma, Ikuyo Nakajima, Atsushi Tajima, Atsuko Abe, Keisuke Sasaki

**Affiliations:** ^1^ Institute of Livestock and Grassland Science National Agriculture and Food Research Organization (NARO) Tsukuba Japan; ^2^ Ehime Research Institute of Agriculture, Forestry and Fisheries Livestock Research Center Seiyo Japan; ^3^ Faculty of Life and Environmental Sciences University of Tsukuba Tsukuba Japan; ^4^ Miyagi Prefectural Livestock Experiment Station Ohsaki Japan; ^5^ Shimane Prefectural Livestock Technology Center Izumo Japan

**Keywords:** cooking method, fattening period, intramuscular fat, temporal dominance of sensations, Wagyu, water‐holding capacity

## Abstract

In this study, we assessed the relative sensory perception of Wagyu beef using temporal dominance of sensations (TDS), which is a dynamic sensory method that captures the “dominance of sensation” throughout food consumption. In addition, we checked the integrity of the TDS by comparing the TDS results with a physicochemical analysis. Strip loins were obtained from 24‐ and 28‐month‐old Japanese Black cattle (“Wagyu”) and were cooked by grilling (*yakiniku*) or boiling (*shabu‐shabu*). Temporal dominance of sensations was then used to evaluate the four types of samples. “Tender and/or soft,” “juicy,” “dry,” “fat melting,” “fat taste,” “umami,” “sweet taste,” and “butter odor” were dominant in at least one of the sample types, with the *yakiniku* cooking method highlighting texture‐ and fat‐related sensory characteristics, and the *shabu‐shabu* cooking method highlighting flavor‐related sensory characteristics. In addition, beef obtained from the 24‐month‐old Wagyu was significantly more “dry” than that of the 28‐month‐old cattle, reflecting their different cooking loss. Temporal dominance of sensations successfully demonstrated the dominant sensory perceptions of Wagyu beef prepared with different cooking methods and fattening periods.

## 1 INTRODUCTION

Japanese Black cattle, or “Wagyu,” are characterized by their ability to deposit very large amounts of intramuscular fat (IMF) (Gotoh et al., 2009; Motoyama, Sasaki, & Watanabe, 2016; Zembayashi, 1994), which results in Wagyu beef having a high tenderness and juiciness (Sasaki, Ooi, et al., 2017b). In addition, the high‐IMF content of Wagyu beef causes it to have a unique sweet and fatty odor that is not detected in the beef produced in United States of America (Matsuishi, Fujimori, & Okitani, 2001) and affects the perception of “umami,” which is one of the five basic tastes (Iida, Saitou, Kawamura, Yamaguchi, & Nishimura, 2015).

To characterize the eating quality of Wagyu beef in more details, it is necessary an understanding of temporal sensory perceptions of Wagyu beef while eating. Various temporal methods have been developed to obtain temporal information. Time intensity is one of the popular temporal methods used to measure the rate, duration, and intensity of one or two sensory attributes and the change in these factors over time (Lee & Pangborn, 1986). The progressive profile method measures the sensory perception of the intensity of specific characteristics and enables, at predefined moments, the monitoring of perceptual intensity changes during product evaluation (Jack, Piggott, & Paterson, 1994). Temporal dominance of sensations (TDS) captures the “dominance of sensations” during food consumption and allows the relative contribution of multiple sensory characteristics to be calculated as a “dominance rate” over time (Pineau et al., 2009). Rodrigues, Souza, Lima, Cruz, and Pinheiro (2018) demonstrated the texture and taste sensations simultaneously and separately on TDS descriptions for cheese. A more recent method is the temporal check‐all‐that‐apply; in this method, panelists are instructed to evaluate the product over time, and constantly check and uncheck the attributes as they are or are not perceived, respectively (Castura, Antúnez, Giménez, & Ares, 2016). Esmerino et al. (2017) compared temporal perceptions of dairy products using TDS, progressive profile, and temporal check‐all‐that‐apply; all three dynamic sensory methods revealed similar temporal sensory profiling of samples.

Sensory perception while eating is a temporal process; however, the perception of several sensory characteristics overlaps at times. Many sensory factors contribute to the Wagyu beef sensory perception. Therefore, a dynamic analysis of the relative contribution of multiple sensory characteristics would help us to understand the complex sensory perception of Wagyu beef. To elucidate the relative importance of the sensory characteristics of Wagyu beef, we predicted that TDS would be a suitable method because the dominance rate of each sensory characteristic is computed during food consumption.

In Japan, Wagyu beef is cooked using various methods, such as “*yakiniku*” (a Japanese‐style meat grilling method), “*shabu‐shabu*” (a Japanese‐style meat boiling method), and “*sukiyaki*” (a Japanese‐style meat grilling and boiling combination method) (Hosking, 2015). It has previously been reported that water bath cooking results in a higher Warner–Bratzler shear force value than belt grill cooking for beef (Obuz, Dikeman, Grobbel, Stephens, & Loughin, 2004) and that the composition of volatile compounds differs between boiled and roast beef (Mottram, 1998). Therefore, since physicochemical factors relate to the sensory characteristics of beef, it is important that the cooking method is considered when assessing the sensory perception of Wagyu beef.

Physical factors that are related to the unique sensory characteristics of Wagyu beef are also affected by the fattening period, as the IMF content of the longissimus muscle gradually increase during the fattening period (Gotoh et al., 2009) and the shear force value of the longissimus muscle decreases between 24 and 32 months of age due to the deposition of IMF (Nishimura, Hattori, & Takahashi, 1999). Thus, the effect of the fattening period on the sensory characteristics of Wagyu beef also needs to be considered.

In this study, we used TDS to examine the sensory perception of Wagyu beef prepared with different cooking methods and fattening periods.

## 2 MATERIALS AND METHODS

### 2.1 Samples

Wagyu beef samples were purchased from the Japan Agricultural Cooperatives‐Shimane. Three Wagyu cattle born in December 2014 comprised the 24‐month‐old group, and three Wagyu cattle born in July or August 2014 were used in the 28‐month‐old group. The ages of 24 and 28 months are considered short‐term and customary fattening periods, respectively, in Japan for Wagyu steers, according to the Japanese feeding standard (NARO, 2008). The Wagyu steers were bred by the Japan Agricultural Cooperatives‐Shimane and raised on the same commercial growing diet with timothy until 12 months of age. Subsequently, Wagyu steers were raised on the same commercial finishing diet with rice straw until slaughtering. The cattle were then slaughtered at the Shimane prefectural animal husbandry Co., Ltd. on 30 November or 8 December 2016. According to the Japan Beef Carcass Grading Standard, two carcasses were graded as A4 and one was graded as A3 among the 24‐month‐old Wagyu steers. The 28‐month‐old Wagyu steers were graded as A3, A4, and A5. Three strip loin subprimals were obtained from separate carcasses in each group. Each portion was wet‐aged at 4°C for 20 days and then divided into three parts. Starting from the head side, these parts were labeled as front, middle, and back. Each part was vacuum‐packed and stored at −30°C. Temporal dominance of sensations assessment was demonstrated on the front parts, and the physicochemical analysis was performed on the middle parts; the back parts were stocked as a reserve.

### 2.2 Physicochemical analysis

Each of the subprimal was thawed in a refrigerator at 4°C for 1 day prior to the physicochemical analysis. Each subprimal part was trimmed, and the longissimus muscle was prepared for physicochemical analysis.

Moisture and IMF content were quantified by normal thermal drying and ether extraction, according to the ISO recommended standards, respectively (1442:1997; ISO, 1997; 1443:1973; ISO, 1973). Moisture and IMF content were measured in two replicate samples from each longissimus muscle, and the average values of moisture and IMF content were recorded for each sample. The melting point of the extracted lipids of IMF was determined using the capillary tube method (Horwitz, 1975).

To measure cooking loss, each longissimus muscle sample was processed into four blocks (2.0 cm × 4.0 cm × 4.0 cm), individually placed in a plastic bag, and immersed in a water bath set at 72°C until they reached an internal temperature of 71°C, which was monitored using a thermorecorder (midi LOGGER GL800; GRAPHTEC) fitted with a type‐K thermocouple. The samples were then cooled by placing the plastic bag in tap running water for 30 min. Weight loss from cooking was then calculated as a percentage of the initial weight before cooking. Cooking loss was measured in four replicate samples from each carcass, following which the average value was calculated.

The Warner–Bratzler shear force (WBSF) was determined as described previously (Sasaki, Ooi, et al., 2017b). Two cooked beef blocks which were used for cooking loss measurement were used for WBSF of each Wagyu steer. Four cores were 1.27 cm (0.5 in.) in diameter, were prepared from each cooked beef block, and sheared perpendicular to the orientation of the muscle fiber using an Instron Universal Testing Machine (Model 5542; Instron Corp.) fitted with a 500N compression load cell with a crosshead speed of 250 mm/min. The average value of four cores was calculated as the WBSF of each cooked block, and the average value of two blocks was recorded as the WBSF for each Wagyu steer.

The texture profile analysis (TPA) of the cooked beef samples was determined as described previously (Caine, Aalhus, Best, Dugan, & Jeremiah, 2003), with a slight modification (Sasaki, Motoyama, et al., 2017a), using an Instron 5542 testing machine attached to a diameter 4.0‐cm disk‐type probe. Two cooked beef blocks, which were used for the cooking loss measurement, were used for the TPA measurement of each Wagyu steer. Four cores were 1.27 cm (0.5 in.) in diameter and 1 cm in height were prepared from each cooked beef block. Each core underwent two cycles of 80% compression along the muscle fiber direction, with a crosshead speed of 60 mm/min using the TPA Test Method Template (ver. 3.0; Instron). The TPA values of “hardness,” “gumminess,” “springiness,” “cohesiveness,” and “chewiness” were calculated (Caine et al., 2003). The average value of four cores was calculated as the TPA parameters of each cooked block, and the average value of two blocks was recorded as the TPA parameters for each Wagyu steer.

A homogenate was used for the analysis of free amino acids, free peptides, inosine monophosphate (IMP), and thiobarbituric acid‐reactive substance (TBARS). A 10‐g portion of each muscle sample was homogenized in 60 ml of ultrapure water. The final volume of the homogenate was adjusted to 100 ml using ultrapure water. To determine the free amino acid content of the muscle, the samples were prepared as described previously (Chikuni et al., 2002). A 5‐ml sample of muscle homogenate was deproteinized using 5 ml of 10% (w/v) trichloroacetic acid, centrifuged at 1,870 g, and incubated at 4°C for 30 min. The supernatant was filtered through a 0.45‐μm nitrocellulose filter, and the free amino acid concentration in the muscle was measured using an amino acid analyzer (L‐8900, Hitachi Ltd.). The amino acids were detected using the ninhydrin method, with detection wavelengths of 440 and 570 nm. Inosine monophosphate was determined using reverse‐phase high‐performance liquid chromatography, as described previously by Ryder (1985). Free oligopeptides were analyzed using the Lowry method, as described previously (Mikami, Nagao, Sekikawa, & Miura, 1995). Thiobarbituric acid‐reactive substance was determined by a spectrophotometric assay, as described previously (Witte, Krause, & Bailey, 1970) with some modification (Mitsumoto, Arnold, Schaefer, & Cassens, 1993).

The fatty acid profile of each beef sample was assessed using gas–liquid chromatography (GLC). Intramuscular fat was extracted from a 1‐g portion of each muscle sample using 10 ml of chloroform:methanol (2:1, v/v). Methyl esters of fatty acids were then prepared using a fatty acid methylation kit (Nacalai Tesque) and were purified with a fatty acid methyl ester purification kit (Nacalai Tesque). A GC‐2010 Plus (Shimadzu) equipped with a flame ionization detector and an URBON HR‐SS‐10 capillary column (0.25 mm × 30 m; Shinwa Chemical Industries) was used for the GLC analysis. The column temperature was held at 140°C for 3 min followed by an increase of 2.5°C/min to 210°C. H_2_ was used as the carrier gas with an inlet pressure of 51.8 kPa and a split ratio of 1:10. The injector and detector temperatures were set at 250°C, and the injection volume was 0.1 μl.

### 2.3 Sample preparation for sensory testing

Each of the subprimal was thawed in a refrigerator set at 4°C for 1 day. For the *yakiniku* cooking method, 4‐mm‐thick disks (4 cm diameter) were taken from each sample and cooked on an electric griddle (KZ‐HP1000; Panasonic) at 180°C for 30 s on each side. For the *shabu‐shabu* cooking method, 2‐mm‐thick disks (4 cm diameter) were taken from each sample and cooked in boiling 1% (w/v) NaCl solution for 30 s, which is similar to the general wet heat cooking method for beef in Japan (Sasaki & Mitsumoto, 2003). Each cooked sample was placed in a polypropylene cup covered with a polyethylene terephthalate lid and was kept warm in a warming cabinet (MHW‐S2; Maruzen Co., Ltd.) set at 70°C until immediately before the TDS assessment.

### 2.4 TDS analysis

#### 2.4.1 Training session

Sixteen staff members from the Institute of Livestock and Grassland Science, National Agriculture and Food Research Organization (NARO) (Ibaraki, Japan), were selected and trained as sensory panel members, as described previously (Sasaki et al., 2012). These individuals were trained to assess the sensory characteristics of beef, including the texture, taste, and odor, and participated in descriptive sensory evaluations of beef (Sasaki, Ooi, et al., 2017b). In addition, they were trained to understand the concept of “dominance of sensations,” which has been defined as “the sensation that triggers most of the attention at a point of time, which may not be the most intense” (Pineau et al., 2009). They were also trained to use the computerized TDS data capture system MagicSense (Taste Technology LLC.) (Watanabe et al., 2017).

#### 2.4.2 Assessment

The TDS assessment sessions were performed in a sensory test room that was maintained at 22°C by an air conditioner. Each of the panel members undertook the tests in an individual booth that was illuminated by red light.

The following 12 sensory characteristics were established for TDS during preliminary sensory sessions: “tender and/or soft,” “tough and/or hard,” “juicy,” “dry,” “fat melting,” “smoothness,” “umami,” “sweet taste,” “fat taste,” “sweet odors,” “butter odors,” and “oily odors.” These sensory characteristics are defined in Table 1.

**Table 1:** Sensory characteristics that were included in the temporal dominance of sensations (TDS) analysis

Classification	Definition
Texture	
Tender and/or soft	Easy to cut a sample into fragments and/or easy to deform a sample
Tough and/or hard	Difficult to cut a sample into fragments and/or difficult to deform a sample
Juicy	Liquids are released from the surface and body of the sample
Dry	Liquids are absorbed from the surface and body of the sample
Fat melting	Feeling of fat melting in the oral cavity
Smoothness	Feeling of smoothness on the surface
Taste	
Umami	Umami taste (*dashi* taste)
Sweet taste	Sweet taste
Fat taste	Taste of fat; different from the texture of fat
Odor (retronasal)	
Sweet odor	Sweet odor, like vanilla or fruit
Butter odor	Fermented milk‐like odor, like diacetyl
Oily odor	Oily, particularly oxidized oil‐like odor

During TDS test, each panelist assessed all four types of beef samples in each session. A Latin square design was used to avoid the effects of serving order. The panelists were instructed to click on the “START” button as soon as they put the beef sample in their mouth. They were then able to successively select the characteristics that most triggered their attention from the 12 sensory characteristics. They could only click on one characteristic at a time but could change this as many times as they liked whenever a new sensation became dominant and were free to choose a characteristic several times. The sample could be swallowed at any time, at which point the “SWALLOW” button was selected. The panelists then continued to evaluate the taste or retronasal odors from the time of swallowing until they disappeared.

Each TDS test lasted for 60 s, and data were recorded every 0.2 s. When no sensation was perceived as dominant, the panelists were instructed to click on the “ABSENCE” button. The TDS test was carried out three times using samples from different carcasses in each session. In total, 46 runs were conducted for each beef sample because one panelist was absent from the second and third sessions (first session: 16 runs; second session: 15 runs; third session: 15 runs).

### 2.5 Data analysis

All statistical analyses were performed using SAS version 9.4 (SAS Institute). The physicochemical data were analyzed using the general linear model procedure in SAS. The effect of fattening period (24 vs. 28 months) on the Wagyu beef characteristics was analyzed using one‐way analysis of variance (ANOVA).

In TDS analysis, we calculated a dominance rate which is defined as the percentage of selections in which a characteristic is dominant at a particular point in time (Pineau et al., 2009). The proportion of the 46 runs in which a given characteristic was assessed as dominant was calculated for each 0.2‐s time interval. These proportions were then transformed by a noniterative smoothing spline using the TRANSREG procedure in SAS and plotted against time to create a “TDS curve” (Pineau et al., 2009). Temporal dominance of sensations curves of all 12 characteristics were depicted on the same graph for each type of beef sample.

The “chance level” and “significance level” were also calculated for each type of beef sample as described previously (Pineau et al., 2009). In this study, the chance level and the significance level for each characteristic were 0.083 and 0.150, respectively. When the dominance rate exceeded the significance level, the characteristic was considered to be dominant.

Temporal dominance of sensations difference curves (Pineau et al., 2009) were also constructed to assess the effects of cooking method and fattening period on the sensory characteristics of Wagyu beef. In this study, a TDS difference curve for the comparison of cooking methods was computed for each fattening period and a TDS difference curve for the comparison of fattening periods was computed for each cooking method.

## 3 RESULTS AND DISCUSSION

### 3.1 Physicochemical analysis

The results of the physicochemical analysis of Wagyu beef strip loin obtained from steer that were 24 and 28 months of age are shown in Table 2. It has been reported that the IMF content of Wagyu gradually increases during the fattening period (Gotoh et al., 2009; Nishimura et al., 1999). However, in the present study, there was no significant difference in the IMF of the Wagyu beef obtained from different‐aged cattle. Previously, Iwamoto, Oka, and Iwaki (2009) reported no differences in the IMF content of Wagyu beef obtained from 20‐ and 24‐month‐old cattle, which is consistent with our results. These data suggest that a 4‐month difference in the duration of the fattening period does not affect the IMF content of Wagyu beef.

**Table 2:** Physicochemical analysis of strip loins obtained from 24‐ and 28‐month‐old Wagyu cattle^a^

Instrumental characteristic	Feeding period (months)		*SE*	*p*‐value^b^
	24	28		
Moisture (g/100 g meat)	47.80	44.95	3.75	NS
Intramuscular fat (g/100 g meat)	37.12	37.08	4.86	NS
Cooking loss (g/100 g meat)	14.28	11.98	0.29	**
Thiobarbituric acid‐reactive substances (nmol malondialdehyde equivalents/g meat)	2.49	2.93	0.12	NS
Warner–Bratzler shear force (N)	18.51	14.12	1.65	NS
Hardness by texture profile analysis (N)	20.69	15.40	1.37	NS
Gumminess by texture profile analysis (N)	4.38	3.00	0.36	NS
Springiness by texture profile analysis (mm)	2.80	2.66	0.03	*
Cohesiveness by texture profile analysis	0.19	0.21	0.01	NS
Chewiness by texture profile analysis (N)	12.24	7.98	0.94	*
Fat melting point (°C)	32.60	30.30	1.40	NS
Taste‐relevant components				
Free glutamate (μmol/g meat)	1.05	0.98	0.14	NS
Free alanine (μmol/g meat)	4.11	3.79	0.19	NS
Total free amino acid (μmol/g meat)	14.88	14.50	0.83	NS
Total free peptide (mg/g meat)	2.43	2.82	0.23	NS
Inosine monophosphate (μmol/g meat)	0.95	1.07	0.27	NS

Note

Values are expressed as least‐squares means.

^a^ *n* = 3 in each group.

^b^ ANOVA: *p < .05; **p < .01; NS, not significant.

Beef obtained from the 24‐month‐old Wagyu had significantly higher levels of cooking loss, springiness, and chewiness than that obtained from the 28‐month‐old cattle (*p* < .05), suggesting that the fattening period affected the water‐holding capacity of the meat during the heating process and its springiness and chewiness. According to a previous study, the IMF content is negatively correlated with the cooking loss (Ueda et al., 2007). However, in the present study, there was no difference in the IMF content between the Wagyu cattle of different ages. It is possible that the high cooking loss of 24‐month‐old cattle was induced by other factors. The majority of the moisture in the muscle is held within the structure of the muscle and muscle cells (Offer & Cousins, 1992). The integrity of skeletal muscle is maintained by intramuscular connective tissues (Nishimura, 2015), and the thermal and mechanical stability of these connective tissues increases with growth (Bailey & Light, 1989; McCormick, 1994; Nishimura, Ojima, Liu, Hattori, & Takahashi, 1996). Thus, the difference of the stability of the intramuscular connective tissues between cattle of different ages may affect the water loss during cooking. On the other hand, the springiness and chewiness of 24‐month‐old cattle were significantly higher than that of 28‐month‐old cattle. The chewiness and springiness by TPA are different between the beef which have the different cooking loss (Palka & Daun, 1999). Thus, high cooking loss may be associated with high chewiness and springiness by TPA in 24‐month‐old cattle.

No significant difference was observed in the moisture, WBSF, and IMP content of the Wagyu beef between cattle of different aged. In high‐marbled beef, the IMF content correlates negatively with moisture (Savell, Cross, & Smith, 1986; Ueda et al., 2007), WBSF (Ueda et al., 2007), and IMP content (Iida et al., 2015). Additionally, no difference was detected in the free amino acids between the Wagyu beef obtained from 24‐ and 28‐month‐old cattle. Our results are consistent with previous studies showing that free glutamate and total free amino acids do not differ between Wagyu cattle of different ages (Iwamoto et al., 2009; Watanabe, Ueda, & Higuchi, 2004). Moreover, there was no significant difference in the fatty acid profiles of the Wagyu beef obtained from 24‐ and 28‐month‐old cattle (Table 3). In a previous study, the fatty acid composition of Wagyu beef obtained from 20‐ and 24‐month‐old cattle showed no differences (Iwamoto et al., 2009), which is consistent with our results. Furthermore, there was no significant difference in the TBARS and fat melting point. Both these parameters are affected by fatty acid composition (Wood et al., 2008). Thus, the absence of any differences in fatty acid composition between the Wagyu cattle of different ages may explain the lack of differences in TBARS and fat melting point.

**Table 3:** Fatty acid compositions of strip loins obtained from 24‐ and 28‐month‐old Wagyu^a^

Fatty acid, %	Feeding period (months)		*SE*	*p*‐value^b^
	24	28		
C14:0 (myristic acid)	2.73	2.52	0.19	NS
C14:1 (myristoleic acid)	2.02	1.66	0.40	NS
C16:0 (palmitic acid)	19.25	20.95	0.59	NS
C16:1 (palmitoleic acid)	5.93	6.05	0.57	NS
C18:0 (stearic acid)	6.72	6.81	0.39	NS
C18:1 (oleic acid)	60.21	57.94	1.60	NS
C18:2, n‐6 (linoleic acid)	3.14	4.07	0.45	NS
C18:3, n‐3 (α‐linolenic acid)	ND^c^	ND		
Saturated fatty acid	28.70	30.28	0.76	NS
Monounsaturated fatty acid	68.16	65.65	0.87	NS
Polyunsaturated fatty acid	3.14	4.07	0.45	NS

Note

Values are expressed as least‐squares means.

^a^ *n* = 3 in each group.

^b^ ANOVA: NS, not significant.

^c^ ND, not detected.

### 3.2 TDS curves

**Figure 1:**
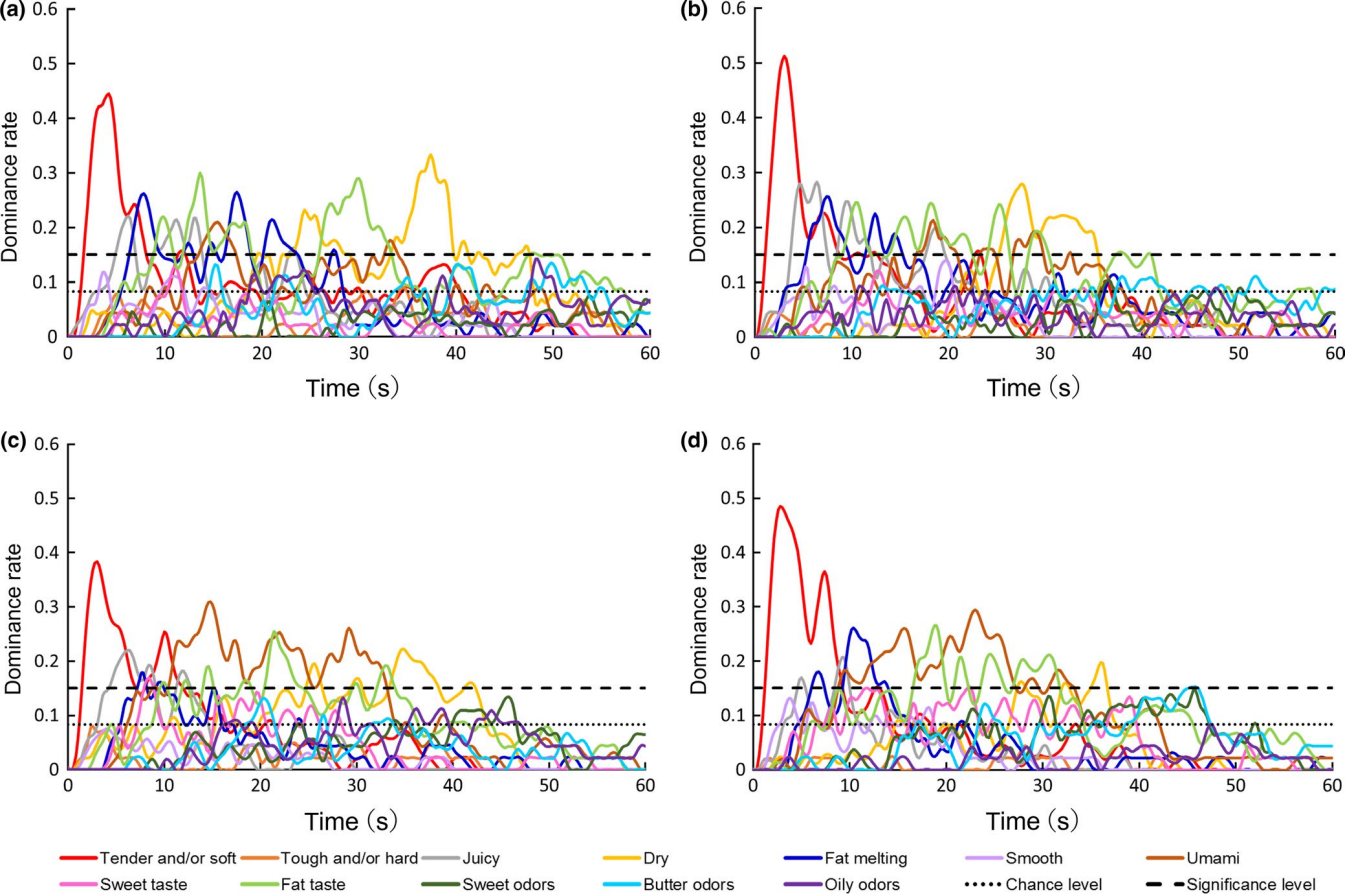
Temporal dominance of sensations (TDS) curves for strip loins of Wagyu beef obtained from (a) 24‐month‐old cattle and cooked using the *yakiniku* method, (b) 28‐month‐old cattle and cooked using the *yakiniku* method, (c) 24‐month‐old cattle and cooked using the *shabu‐shabu* method, and (d) 28‐month‐old cattle and cooked using the *shabu‐shabu* method

The TDS curves for each of the 12 sensory characteristics that were assessed in each of the four different types of beef samples (two cooking methods and fattening periods) are shown in Figure 1. When the dominance rate of a particular sensory characteristic exceeded the significance level of 0.150, it was considered dominant.

In all four sample types, “tender and/or soft,” “juicy,” “dry,” “fat melting,” “fat taste,” and “umami” became dominant at some point after the start of eating (Figure 1). Among the *shabu‐shabu* samples, “sweet taste” exceeded the significance level in cattle of both ages (Figure 1c,d), while “butter odor” exceeded the significance level in the samples obtained from 28‐month‐old Wagyu (Figure 1d). Therefore, these characteristics are considered to contribute to the sensory perception of Wagyu beef. It was reported that “tenderness,” “juiciness,” and “fattiness” are unique sensory characteristics of high‐IMF Wagyu beef (Iida et al., 2015), and the present TDS analysis also demonstrated the significant contribution of these sensory characteristics.

“Umami” is defined as the taste of glutamate and nucleotides such as IMP and guanosine monophosphate (Lindemann, Ogiwara, & Ninomiya, 2002) and is generally associated with a “brothy” taste (Maga, 1994). “Umami” has been reported as the major sensory characteristic of chicken (Fujimura et al., 1995) and pork (Watanabe et al., 2017) but its relative contribution to the sensory perception of beef has not previously been demonstrated. We hypothesized that “umami” would be difficult to perceive in high‐IMF beef such as Wagyu because the taste‐active component of “umami” is water soluble and is present at a lower concentration in Wagyu than in lean beef (Sasaki, Ooi, et al., 2017b). However, contrary to this hypothesis, our results indicated that “umami” was the dominant sensory characteristic of Wagyu beef.

Interestingly, “sweet taste” also became dominant in the *shabu‐shabu* samples. There has been little research on the components that contribute to the sweet taste of meat, but alanine has been identified as a taste‐active component of stewed beef juice (Schlichtherle‐Cerny & Grosch, 1998). Therefore, it is possible that alanine also contributed to the perception of “sweet taste” in the Wagyu beef, although other sweet taste components may have also contributed to this.

Analysis of the TDS curves for all four types of beef samples (Figure 1) showed that “tender and/or soft” was mainly perceived in the first 10 s of eating, while “juicy,” “fat taste,” “fat melting,” “umami,” and “sweet taste” overlapped at around 5–30 s, and “dry,” “fat taste,” and “butter odor” became dominant at 30–50 s. In general, food consumption begins with the intake of food, followed by a succession of chewing cycles and swallowing (Bostman, Van Der Bilt, Abbink, & Van Der Glas, 2004). The chewing cycles serve to break down the food (Carpenter & Blissett, 2017) and the chewing process triggers the secretion of saliva, which acts as a solvent for taste‐active components (Matsuo, 2000) and affects the perception of juiciness (Aaslyng, Bejerholm, Ertbjerg, Bertram, & Andersen, 2003). Finally, the retronasal aroma is mainly perceived when a small volume of air is exhaled immediately after swallowing (Land, 1994). The TDS curves provided a good overall description of the major sensory characteristics of Wagyu beef during the consumption process.

### 3.3 TDS difference curves for cooking methods

**Figure 2:**
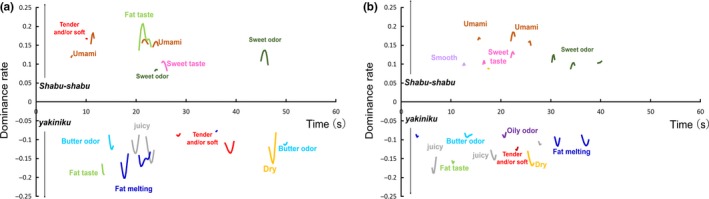
Temporal dominance of sensations (TDS) difference curves for strip loins of Wagyu beef cooked using the *yakiniku* and *shabu‐shabu* methods obtained from (a) 24‐month‐old cattle and (b) 28‐month‐old cattle

The TDS difference curves for the characteristics of Wagyu beef cooked with the *yakiniku* and *shabu‐shabu* methods are shown in Figure 2.

Texture‐related sensory characteristics such as “tender and/or soft,” “fat melting,” “juicy,” and “dry” were highlighted by the *yakiniku* method of cooking (Figure 2). It was reported that grilled beef has a significantly higher IMF content than poached beef (Iida, Horie, & Nishimura, 2014), so the texture‐related characteristics that are associated with high levels of IMF may be more prominent in *yakiniku* Wagyu beef. In addition, it was reported that the effect of “tenderness” and “juiciness” on the predict beef preference was slightly different between *yakiniku* and *shabu‐shabu* methods (Polkinghorne, Nishimura, Neath, & Watson, 2011). This difference might be due to the difference of relative sensory perception of “tenderness” and “juiciness” between *yakiniku* and *shabu‐shabu* methods.

Taste‐related sensory characteristics such as “umami” and “sweet taste” exhibited a significantly higher dominance rate in meat cooked with the *shabu‐shabu* method rather than the *yakiniku* method (Figure 2). Poached beef had significantly lower IMP and total free amino acid contents than grilled beef (Iida et al., 2014), which suggests that this dominance of “umami” and “sweet taste” in the *shabu‐shabu* samples did not result from a difference in content of taste‐active components. The *shabu‐shabu* samples were thinner than the *yakiniku* samples because they were prepared in the traditional way for this cooking method. Moreover, the IMF content of the beef was lower in the poached sample than in the grilled sample (Iida et al., 2014). Therefore, this difference in thickness and IMF content may have led to a low perception of texture‐related sensory characteristics in the *shabu‐shabu* samples, which, in turn, may have highlighted the perception of the taste‐related sensory characteristics.

In terms of the retronasal odors, “butter odor” was highlighted in the *yakiniku* samples (Figure 2). It was reported that the volatile component of the butter‐like odor, such as diacetyl and acetoin, was higher in roasted Wagyu beef than in poached Wagyu beef (Taguchi, Takahashi, Yasuhara, Asano, & Iida, 2013). Therefore, this enhanced “butter odor” in the *yakiniku* samples was likely induced by the higher concentration of these volatile components. By contrast, “sweet odor” was highlighted in the *shabu‐shabu* samples. It was demonstrated that lactones contribute to the “sweet odor” of Wagyu beef (Matsuishi et al., 2004) and Wagyu beef had the strongest unique sweet odor when boiled at 80°C compared with 40, 60, and 100°C (Matsuishi et al., 2001). Therefore, the “sweet odor” may have been more perceptible in the *shabu‐shabu* samples than in the *yakiniku* samples because the *yakiniku* cooking method used too high a temperature.

As described above, there are differences in the dynamic sensory perception of texture, taste, and odor in Wagyu beef samples depending on the cooking method. It has previously been reported that the perception of both texture and flavor sensory characteristics was different between the oven‐cooked and fried polenta sticks by TDS method (Monaco, Miele, Volpe, Masi, & Cavella, 2016). Thus, the effect of the cooking method on sensory perception during food consumption should be considered in various sensory characteristics.

### 3.4 TDS difference curve for fattening periods

**Figure 3:**
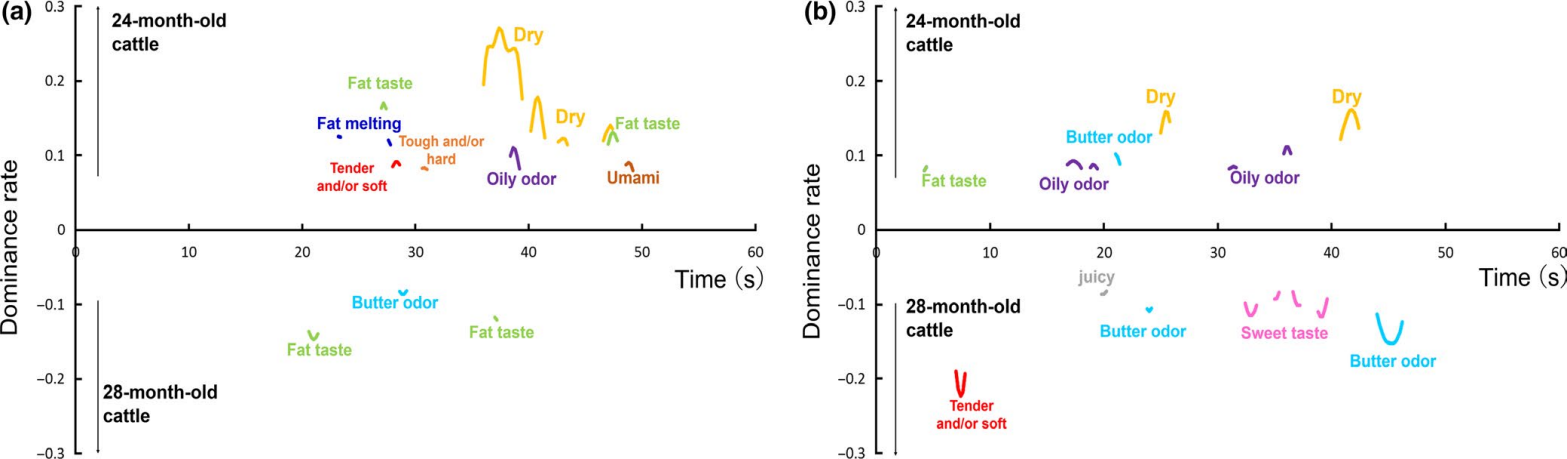
Temporal dominance of sensations (TDS) difference curves for strip loins of Wagyu beef obtained from 24‐ and 28‐month‐old cattle that were cooked using the (a) *yakiniku* method and (b) *shabu‐shabu* method

The TDS difference curves for the characteristics of Wagyu beef obtained from 24‐ and 28‐month‐old cattle are displayed in Figure 3.

“Dry” had a significantly higher dominance rate in Wagyu beef obtained from 24‐month‐old cattle than in that obtained from 28‐month‐old cattle with both cooking methods, although this difference was detectible for longer with the *yakiniku* samples (at around 35–50 s; Figure 3a) than with the *shabu‐shabu* samples (Figure 3b). Many studies have reported a positive relationship between IMF content and “juiciness” (Blumer, 1963; Fernandez, Monin, Talmant, Mourot, & Lebret, 1999; Font‐i‐Furnols, Tous, Esteve‐Garcia, & Gispert, 2012; Thompson, 2004). However, in the present study, there was no difference in IMF content between the Wagyu beef obtained from the different‐aged cattle (Table 2). The water‐holding capacity has also been shown to affect the juiciness of meat, with the juiciness that is experienced at the start of the chewing process is decreased as the cooking loss increases in pork (Aaslyng et al., 2003), and a high internal temperature induces a high cooking loss in beef, which decreases both the initial and sustained juiciness (Cross, Stanfield, & Koch, 1976). In the present study, Wagyu beef obtained from 24‐month‐old cattle had a significantly higher cooking loss than that obtained from 28‐month‐old cattle (*p* < .01; Table 2). Therefore, the enhanced perception of “dry” in the Wagyu beef obtained from younger cattle seems to have resulted from water loss during the cooking process.

The dominance rates of “tender and/or soft,” “tough and/or hard,” “fat taste,” “fat melting,” “sweet taste,” “umami,” “butter odor,” and “oily odor” also significantly differed between the two fattening periods for a short time (Figure 3). However, there was no significant difference in the physicochemical parameters or fatty acid profiles that related to these sensory characteristics between these two types of Wagyu beef. It should be noted that the instrumental parameters were measured only in raw beef samples. Therefore, further research is required to investigate differences in the physicochemical properties and volatile components of cooked Wagyu beef samples obtained from cattle of different ages.

## 4 CONCLUSION

In this study, we used TDS to investigate the relative sensory perception of Wagyu beef prepared with different cooking methods and fattening periods. We found that “tender and/or soft,” “juicy,” “dry,” “fat melting,” “fat taste,” “umami,” “sweet taste,” and “butter odor” are the major sensory characteristics of Wagyu beef, and that there are differences in the dynamic sensory perception of meat samples depending on both the cooking method and fattening period. Thus, TDS revealed novel information that helped us to understand the complex sensory characteristics of Wagyu beef.

In the present study, trained panelists showed the dynamics of dominant sensations of Wagyu beef, but we did not demonstrate the consumer's perception. In the future, we will investigate the consumer's perceptions of Wagyu beef obtained from different fattening periods and cooking methods and compare with our TDS results of trained panelists. In addition, consumer's preference and acceptability of meat and meat products are affected by various sensory factors. Therefore, to strategically market Wagyu beef, it is important to investigate the consumer's acceptance of Wagyu beef. Combination of these consumer assessments with the TDS results will contribute toward developing high‐quality Wagyu beef and its marketing strategies.

## CONFLICT OF INTEREST

The authors declare that they do not have any conflict of interest.

## ETHICAL APPROVAL

This study does not involve animal testing. In sensory evaluation, each panelist was informed of the safety of the beef samples and their rights of subjects and then each consented to participate as a sensory panelist in the experiments. This informed consent has been obtained and documented. In addition, sensory evaluations were conducted in an anonymous fashion. These procedures were approved in accordance with the Implementation Guidelines for Research Involving Human Subjects in Institute of Livestock and Grassland Science, National Agriculture and Food Research Organization, Japan.
